# Altered Cell Cycle Gene Expression and Apoptosis in Post-Implantation Dog Parthenotes

**DOI:** 10.1371/journal.pone.0041256

**Published:** 2012-08-15

**Authors:** Jung Eun Park, Min Jung Kim, Seung Kwon Ha, So Gun Hong, Hyun Ju Oh, Geon A. Kim, Eun Jung Park, Jung Taek Kang, Islam M. Saadeldin, Goo Jang, Byeong Chun Lee

**Affiliations:** 1 Department of Theriogenology & Biotechnology, College of Veterinary Medicine, Seoul National University, Seoul, Republic of Korea; 2 Life Science of R&D Center, SK chemicals, Bundang-gu, Seongnam-si, Gyeonggi-do, Seoul, Republic of Korea; 3 Department of Physiology, Faculty of Veterinary Medicine, Zagazig University, Zagazig, Sharkia, Egypt; State Key Laboratory of Reproductive Biology, Institute of Zoology, Chinese Academy of Sciences, China

## Abstract

Mature oocytes can be parthenogenetically activated by a variety of methods and the resulting embryos are valuable for studies of the respective roles of paternal and maternal genomes in early mammalian development. In the present study, we report the first successful development of parthenogenetic canine embryos to the post-implantation stage. Nine out of ten embryo transfer recipients became pregnant and successful *in utero* development of canine parthenotes was confirmed. For further evaluation of these parthenotes, their fetal development was compared with artificially inseminated controls and differentially expressed genes (DEGs) were compared using ACP RT-PCR, histological analysis and immunohistochemistry. We found formation of the limb-bud and no obvious differences in histological appearance of the canine parthenote recovered before degeneration occurred; however canine parthenotes were developmentally delayed with different cell cycle regulating-, mitochondria-related and apoptosis-related gene expression patterns compared with controls. In conclusion, our protocols were suitable for activating canine oocytes artificially and supported early fetal development. We demonstrated that the developmental abnormalities in canine parthenotes may result from defective regulation of apoptosis and aberrant gene expression patterns, and provided evidence that canine parthenotes can be a useful tool for screening and for comparative studies of imprinted genes.

## Introduction

Parthenogenesis is the process by which oocytes can develop without fertilization, and the resulting parthenogenetic embryos, called parthenotes, carry only maternal chromosomes [1]. Mature oocytes can be parthenogenetically activated by a variety of electric, mechanical or chemical stimulation methods in mammals. These embryos can be valuable for functional assays of oocyte developmental competence and allow studies of the respective roles of paternal and maternal genomes in early mammalian development [2], [3], [4], [5]. Furthermore, parthenogenesis has the potential to produce pluripotent cells and may be useful for basic scientific studies as well as novel therapeutic applications [1], [6], [7], [8].

For normal development of offspring in mammals, precise regulation of maternal and paternal genomes is required [9]. Several attempts have been made to understand detailed rules of parental-origin specific gene expression by comparing development of uniparental embryos with their biparental counterparts [4], [10]. In general, mammalian parthenotes fail to reach term development. Morphological analysis of mouse parthenotes showed that the developmental failure occurred at different time points during early post-implantation development, and a critical influence of genomic imprinting on the regulation of early development was reported [11], [12], [13]. In addition, candidates for imprinting have been predicted by comparing gene expression patterns among parthenogenetic, androgenetic and normal fertilized embryos [14], [15]. Although parthenogenetic embryos can implant *in vivo* in several species including mouse, pig, rabbit and sheep [16], [17], [18], [19], few detailed experiments have been conducted to explore underlying mechanisms of perturbed post-implantation development. As a result, critical events leading to the developmental failure of parthenotes have not been fully identified despite the need to elucidate them for a basic understanding of mammalian development.

The dog is an emerging model for human disease, particularly for the study of genetic association of complex traits, assisted by the development of an invaluable genome resource for canine molecular genetics [20], [21]. In this study, we report the first successful post-implantation development of parthenogenetic canine embryos. In order to further understand the molecular basis for embryo development *in utero*, we performed detailed profiling of differentially expressed genes (DEGs) in canine parthenotes and in normally fertilized fetuses. In previous studies, several methodologies such as cDNA microarrays, serial analysis of gene expression (SAGE), suppression subtractive hybridization (SSH) and annealing control primer (ACP) based RT-PCR were utilized for screening of DEGs [22], [23], [24], [25], [26]. The latter method uses ACPs that specifically target sequence hybridization to the template via a polydeozyinosie [poly(dI)] linker and allows only genuine products to be amplified [24], [27]. It has high reproducibility because of the high annealing specificity of the ACPs and is a simple, easy and cost efficient method for identifying DEGs [28], [29]. Accordingly, we applied this technique to explore differentially expressed genes in parthenogenetic and normally fertilized fetuses.

We investigated the ability of parthenogenetic canine embryos to implant *in vivo*, and the molecular basis for development of parthenotes *in utero* by detailed profiling of DEGs, histological examinations and gene expression analyses on canine parthenotes and age-matched control fetuses derived from normal fertilization *in vivo* to gain insights into the underlying mechanisms of impaired fetal development.

## Materials and Methods

### Chemicals

Unless otherwise indicated, chemicals were purchased from Sigma-Aldrich Corp. (St. Louis, MO, USA).

### Ethics statement

All animal studies were conducted in accordance with recommendations described in “The Guide for the Care and Use of Laboratory Animals” published by the Institutional Animal Care and Use Committee (IACUC) of Seoul National University. The protocol was approved by the Committee on the Ethics of Animal Experiment of the Seoul National University (approval number SNU-090508-5), and dog care facilities and the procedures performed met or exceeded the standards established by the Committee for Accreditation of Laboratory Animal Care at Seoul National University. All surgery was performed under isoflurane anesthesia, and every effort was made to minimize suffering.

### Production of canine parthenotes

In this study, mixed breed dogs aged 1.5–3 years with various reproductive histories were used. *In vivo* matured dog oocytes were recovered from anesthetized female dogs by laparotomy. The ampullary portion of the oviduct was accessed and oocytes were recovered by flushing approximately 72 h after ovulation and prepared as previously described [30], [31], [32], [33]. Cumulus cells from *in vivo*-matured canine oocytes were removed by repeated pipetting in 0.1% (v/v) hyaluronidase (from bovine testis) in Hepes-buffered TCM-199 (Invitrogen, Carlsbad, CA, USA) supplemented with 2 mM NaHCO_3_, 5 mg/mL BSA (Invitrogen) and a 1% (v/v) mixture of penicillin and streptomycin. Chemical activation was induced by incubating the denuded oocytes in modified synthetic oviductal fluid (mSOF) containing 10 µM calcium ionophore for 4 min, followed by 4 h of culture in mSOF supplemented with 1.9 mM 6-dimethylaminopurine overlaid with mineral oil at 39°C in a humidified atmosphere of 5% O_2_ and 5% CO_2_ and 90% N_2_ [34], [35]. Within 4 h after activation, parthenogenetic embryos were surgically transferred into the oviducts of recipients in which estrus was naturally synchronous [30], [31], [32], [33].

### Artificial insemination

Artificial insemination (AI) was performed 72 h after ovulation by surgical intrauterine insemination [36]. A recipient dog in natural estrus was placed under general anesthesia and freshly collected semen was introduced into both uterine horns. The collected semen was centrifuged at 750× g for 5 min and the total volume of inseminated semen was adjusted to 1 ml. Semen with sperm motility of at least 80% and total ejaculated sperm ≥200×10^6^ sperm/ml were used for this procedure after conventional evaluation by light microscopy using a Makler counting chamber (ZDL, Inc., Lexington, USA) [36].

### Pregnancy diagnosis and sample collection

Pregnancies were detected 23 to 25 days after AI or embryo transfer using a MyLab30 Gold Ultrasound Scanner (Esaote SpA, Genova, Italy) with an attached 7.0 MHz linear-array transducer. Pregnancy was monitored ultrasonographically after the initial confirmation [30], [31], [32], [37]. The conceptuses derived from the AI group (control) and parthenogenetically activated embryos (PA group) were obtained from recipients on days 28, 30 or 32 of pregnancy, and were analyzed for their size of sac, gross external morphology and weight. The fetuses were then washed in Ca^2+^- and Mg^2+^-free PBS (Invitrogen) and stored at −80°C for gene expression analysis and immersed in 10% neutral buffered formalin for histological analysis. Corpora lutea (CL) were counted in the right and left ovaries by opening the thin part of the ovarian bursa during the AI group sampling. The CL count was regarded as equal to the number of ovulated oocytes.

### RNA extraction and ACP-based GeneFishing PCR

Total RNA was extracted from whole fetuses recovered at 28, 30 and 32 days of pregnancy using the easy-spin™ Total RNA Extraction Kit (Intron, Kyunggi, Korea) according to the manufacturer's protocol. Total RNA from each sample was incubated with 2 µL of dT-ACP1 (10 µM, GeneFishing™ DEG kits, Seegene, Seoul, Korea) at 80°C for 3 min after which the reverse transcriptation (RT) reaction was performed using 2 µL of 10× reaction buffer (Invitrogen), 5 mM MgCl_2_, 1 mM DTT, 1 mM of each dNTP, 40 U of RNase inhibitor, and 200 U of Superscript III reverse transcriptase (Invitrogen) in a 20 µL reaction. The reaction mixture was incubated at 42°C for 90 min and then at 94°C for 2 min. The cDNAs were diluted by the addition of 80 µL of ultra-purified water and then subjected to second-strand cDNA synthesis by random PCR amplification using dT-ACP2 and one of 20 arbitrary ACPs (GeneFishing™ DEG kits) as primers. The PCR protocol for second-strand synthesis was one cycle at 94°C for 1 min, 50°C for 3 min and 72°C for 1 min. After second-strand DNA synthesis was completed, the second-stage PCR amplification protocol was 40 cycles of 94°C for 40 sec, 65°C for 40 sec and 72°C for 40 sec, followed by a 5 min final extension at 72°C [27], [28], [38]. The amplified PCR products were separated in 2% agarose gels and stained with RedSafe™ Nucleic Acid Staining Solution (Intron).

### DNA sequencing and BLAST analysis

Differentially expressed PCR products were gel purified (QIAquick PCR purification kit; QIAGEN, Valencia, CA, USA), and DNA strands were directly sequenced (Macrogen, Seoul, Korea; http://www.macrogen.com) using a custom-synthesized primer (5′-CTGTGAATGCTGCGACTACGA-3′). The identity of each product was confirmed by sequence homology analysis using the Basic Local Alignment Search Tool (BLAST) at the National Center for Biotechnology Information (NCBI) GenBank (http://blast.ncbi.nlm.nih.gov/).

### RT-PCR Quantification

To confirm the results of the ACP RT-PCR analysis and to determine the relative abundance of target sequences, mRNA from each sample was subjected to real-time RT-PCR using specific primers for 3 selected DEGs and genes related to apoptosis, autophage and growth (Table 1). Sequence specific primers were designed to amplify products with lengths ranging from 70 to 186 bp. Standard cDNA synthesis by reverse transcription of the RNA was then performed using the Oligo (dT)_20_ primer and the Superscript III reverse transcriptase enzyme (Invitrogen). Real-time PCR was carried out with a 7300 Real Time PCR system (Applied Biosystems, Foster City, CA, USA) using the DNA-binding dye SYBER Green I (Code RRO41A, Takara, Shiga, Japan) for the detection of PCR products. In order to quantify specific gene expression, the mRNA level in each sample was calculated relative to beta-actin. The relative quantification of gene expression was analyzed by the 2^−ddCt^ method [39]. The sizes of PCR products were confirmed by gel electrophoresis on a standard 1.2% agarose gel stained with Redsafe™ (Intron) and visualized by exposure to ultraviolet light.

**Table 1:** List of sequence-specific primers used for real-time PCR.

Gene	Description	Primer sequences (5′→3′)	GenBank No.	Product size (bp)
ACTB	beta-actin	F-GCTACGTCGCCCTGGACTTC	NM_001003349	86
		R-GCCCGTCGGGTAGTTCGTAG		
NSEBP1	Nuclease sensitive element	F- CCGAGGTCACAAGGACCACATCCA	XM_843567.1	186
	binding protein 1	R- ACTAGCGAGAATGGCGGGACG		
Cyclin D2	G1/S-specific cyclin D2	F- CGAGCACATCCTTCGGAAGCTGC	XM_849493.1	147
		R- GCTCCCACGCTTCCAGTTGCAA		
COI	Cytochrome oxidase subunit I	F- TCGTAACCGCCCATGCTTTCG	XM_850384.1	110
		R- TGCCATGTCCGGAGCACCAA		
BAX	BCL2-associated X protein	F-ACTTTGCCAGCAAACTGGTG	NM_001003011	88
		R-AGGAAGTCCAGTGTCCAGCC		
BCL2	B-cell CLL/lymphoma 2	F-TGAGTACCTGAACCGGCATC	NM_001002949.1	100
		R-GTCAAACAGAGGCTGCATGG		
BCL2L1	BCL2-like 1	F-ACTGTGCGTGGAGAGCGTAG	NM_001003072.1	77
		R-TCAGGTAAGTGGCCATCCAA		
TP53	Tumor protein p53	F-ATGGGAGGCATGAACCGGCG	NM_001003210.1	109
		R-CGGGACAGGCACAAACGCGT		
CASP3	Caspase 3	F-GCGGAAACCCACGGGGTTCG	NM_001003042.1	79
		R-CGGATGCGAGCCCGGGAAAG		
CASP8	Caspase 8	F-ACAAGGGCATCATCTATGGCTCTGA	NM_001048029.1	70
		R-CCAGTGAAGTAAGAGGTCAGCTCA		
CASP9	Caspase 9	F-TCAGTGACGTCTGTGTTCAGGAGA	NM_001031633.1	97
		R-TTGTTGATGATGAGGCAGTAGCCG		
ATG5	Autophagy protein 5	F-TCGTCCTGTGGCTGCAGATGGA	XM_849201.1	158
		R-CACTCAGCCACTGCAGAGGTGT		
BECN1	Beclin 1	F-GTGAGCTTCGTGTGCCAGCGT	XM_537634.2	119
		R-TTCACCTGGGCTGTGGCAAGT		
MAP1LC3B	Microtubule-associated proteins	F-AACGGAGGTTGGGGTGGGAAGC	XM_536756.2	94
	1A/1B light chain 3	R-ATGTGGACAGATGCATGCAGCGG		
IGF2	Insulin-like growth factor 2	F-TCTGGAGACCTACTGTGCCA	XM_540785.2	126
		R-TGCTTCCAGGTGTCGTATTG		
IGF2R	Insulin-like growth factor 2	F-AGGGTGAGGAGGTCAGGTTT	MN_001122602.1	106
	receptor	R-CGCTTTTAAAGTGAGGGCTG		

### Histological analysis and immunohistochemistry

For histological analysis, healthy appearing AI and PA fetuses were selected and trimmed by sagittal section. The trimmed tissues were immersed in 10% neutral buffered formalin and changed into Bouin's fluid for 24 h. After fixation, the tissues were transferred to 70% ethanol and kept there until processed. The tissues were embedded in paraffin wax and sections of 5 µm were cut and stained with H&E for overall morphological evaluation.

For assessing cell proliferation, apoptosis and autophage, fetal tissues were mounted on positively charged slides (Superfrost/Plus slide, Erie Scientific Company, Portsmouth, NH, USA). Endogenous peroxidase was quenched with 3% hydrogen peroxide for 10 min at room temperature. Slides were washed with distilled water for 5 min and placed in plastic Coplin jars containing sodium citrate buffer (pH 6.0). The jars were heated in a domestic microwave oven at the highest setting (800 W) for 30 min and then allowed to cool for 15 min. Primary antibodies were diluted in antibody diluents (Dako, Glostrup, Denmark): polyclonal rabbit anti-caspase 8 (CASP8, 1∶50, abcam, Cambridge, UK), polyclonal rabbit anti-Fas-associated death domain-containing protein (FADD, 1∶50, abcam), polyclonal rabbit anti-LC3A/B (1∶50, abcam), polyclonal rabbit anti- extracellular signal-regulated kinase 1 (ERK1, 1∶50, abcam) and monoclonal mouse anti-proliferating cell nuclear antigen (PCNA, 1∶200, Dako). Then, the slides were incubated with antibody overnight at 4°C in a humidified chamber. After three washes with 0.1% Tween 20 in Tris buffer (pH 7.4), the sections were flooded with dextran polymer (Envision kit, Dako) and incubated for 30 min at room temperature and washed three times with Tris buffer (pH 7.4). The final reaction was produced by immersing the sections in a solution of 3,3′-diaminobenzidine (DAB) for 1 min at room temperature. The sections were lightly counterstained with Real Hematoxylin (Dako) and blued in 0.2% ammonia water.

### Statistical analysis

The data from all experiments were analyzed by Student's *t*-test using a statistical analysis system program (SAS Institute, version 9.1, Cary, NC, USA). Differences among the groups were determined using Tukey's Multiple Range Test, and *P*-values of less than 0.05 were considered significant. The values presented are mean ± SEM unless otherwise stated.

## Results

### Post-implantation development of canine parthenotes

One hundred twenty-three PA embryos were surgically transferred into 10 recipients. For comparative purposes, control fetuses were generated by AI in 3 dogs. The pregnancy rate of the AI group was 100% based on the number of inseminated dogs and 79.5% based on the number of ovulated oocytes which was calculated from the number of CL (Table 2). The PA group pregnancy rate was 90% based on the number of recipients, however the proportion of PA fetuses to transferred embryos was 37.4% (Table 3) which was significantly lower than the AI group (79.5%, p<0.05). Various stages of *in vivo* matured oocytes including early mature, mature and moderately aged were used for parthenogenetic activation, and unlike results reported for SCNT embryos [31], pregnancy was achieved after embryo transfer regardless of the oocyte stage used for activation.

**Table 2:** Postimplantation development following artificial insemination in dogs.

Trial No.^a^	Pregnancy	No. of corpora lutea	No. of implantations	Implantation rate (%^c^)
1	+	14	8	57.1
2	+	12	11	91.7
3	+	13	12	92.3
Total	100%^b^		31	79.5

a Artificial insemination was performed approximately 72 h after serum progesterone concentration reached 4.0–7.5 ng/mL.

b Percentage based on total number of artificially inseminated dogs.

c Percentage based on total number of corpora lutea.

**Table 3:** Post-implantation development of canine parthenogenetic embryos.

Trial No.	No. of oocyte donors	Oocyte status^a^	No. of oocytes flushed	Pregnancy	No. of transferred embryos	No. of implantations	Implantation rate (%^c^)
1	1	Moderate aging	14	+	14	13	92.9
2	1	Mature	7	+	6	5	83.3
3	1	Early mature	10	+	10	2	20
4	1	Early mature	10	+	10	2	20
5	2	Early aging	2	+	8	5	62.5
		mature	6				
6	2	Immature	9	−	22	0	0
		mature	13				
7	2	Mature	9	+	16	4	25
		Mature	7				
8	1	Mature	10	+	10	4	40
9	1	Mature	13	+	13	6	46.2
10	2	Early mature	5	+	14	5	35.7
		mature	9				
Total	14		124	90%^b^	123	46	37.4

a Status *of in vivo* matured canine oocytes flushed from oviducts approximately 72 h after serum progesterone concentration reached 4.0–7.5 ng/mL. Denuded oocytes with 0, 15, 25, 30, and more than 40 µm width of the perivitelline space were categorized as immature, mature, early, moderate and severely aged oocytes, respectively. Reference data from Jang et al [31].

b Percentage based on total number of recipient dogs.

c Percentage based on total number of parthenogenetic embryos transferred.

**Figure 1:**
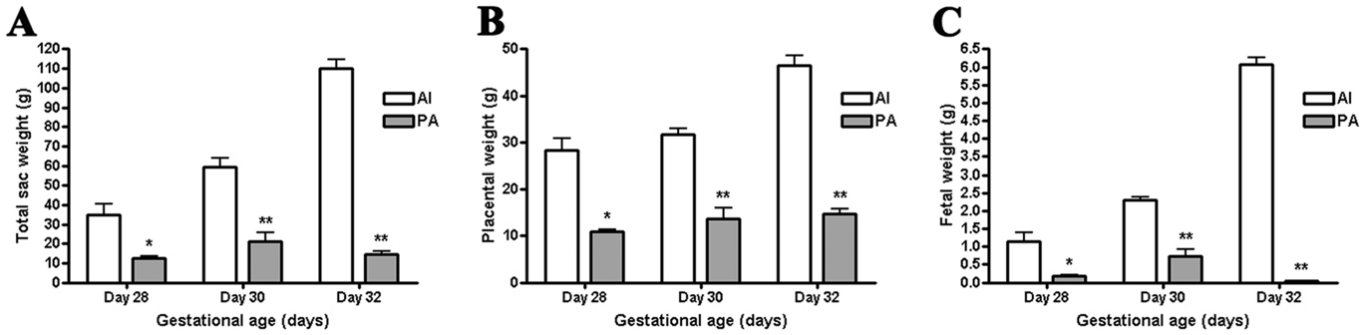
Comparison of fetal and placental growth in controls and canine parthenotes. Measured (A) total sac weight, (B) placental weight, and (C) fetal weights are shown as a function of gestational age. Data presented as mean ± SEM of at least five replicates. ^*, **^ Significant difference between the AI and PA groups at each gestational age (^*^ P<0.05, ^**^ P<0.001).

**Figure 2:**
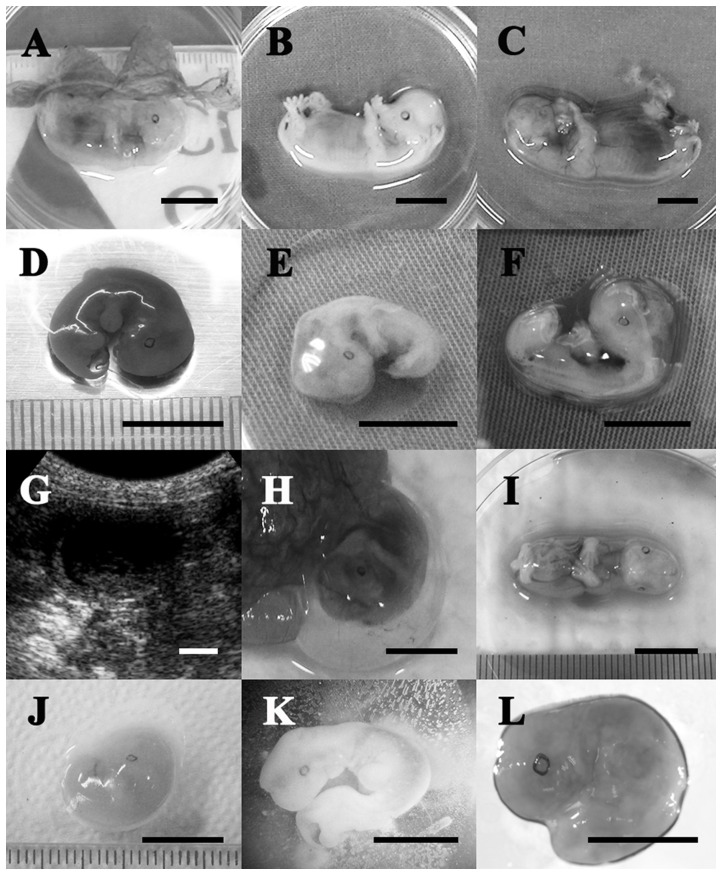
Comparison of control and parthenogenetic canine fetuses. (A–C) The relative size and gross morphology of a control fetus on days 28, 30 and 32 of pregnancy, respectively. (D–F) Parthenogenetic fetuses on day 28 of pregnancy. (G) *In utero* ultrasonograph of parthenogenetic fetuses on day 29 of pregnancy. (H, I) A parthenogenetic fetus recovered on day 30 of pregnancy. Note that the vascular system is well developed and the external morphology is similar to the control. (J) Another small parthenogenetic fetus recovered on day 30 of pregnancy. (K, L) Parthenogenetic fetuses recovered on day 32 of pregnancy. All scale bars represent 10 mm.

To evaluate fetal development of the canine parthenotes, PA fetuses and placental membranes were obtained from recipients and compared with those of similarly aged fetuses from normally fertilized embryos (Fig. 1). The weights of PA fetuses and placentas recovered from uteri at days 28, 30 and 32 of pregnancy were significantly lower than those of the AI group (P<0.05). As shown in Fig. 2, all the recovered parthenotes were able to develop to the stages of limb-bud formation, but much smaller than the control and they probably ceased developing earlier than day 28 of pregnancy. The appearance of some recovered fetuses was comparable to that of the AI group (Fig. 2H and 2I), but the small and degenerating PA fetuses had several unclassifiable and diverse anomalies.

### Identification of DEGs in AI and PA fetuses

**Figure 3:**
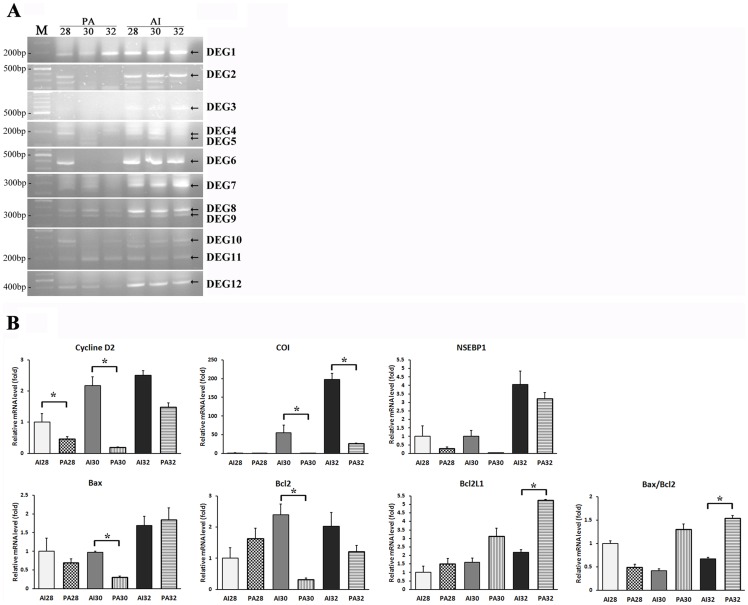
DEGs amplified by ACP-based RT-PCR derived from control and parthenogenetic fetuses and confirmation by real time RT-PCR. (A) Total RNA of the parthenogenetic and control fetuses on days 28, 30 and 32 of pregnancy was used for ACP-based RT-PCR using arbitrary ACP primers (n = 3). Arrows indicate the PCR products of DEGs that were sequenced for gene identification. The twelve sequenced genes were designated as DEG1–12, respectively. (B) Messenger RNA expression of nuclease sensitive element binding protein 1 (NSEBP1, DEG1), G1/S-specific cyclin D2 (Cyclin D2, DEG2), cytochrome oxidase subunit I (COI, DEG7), BAX, BCL2, BCL2L1, and BAX/BCL2 ratio. Data presented as mean ± SEM of at least three replicates. The asterisk denotes a probability value of <0.05.

The PA fetuses on day 28, 30 and 32 of pregnancy were compared to those of the AI control group to identify and isolate the DEGs using a combination of 20 arbitrary ACP primers and two oligo dT primers (dT-ACP1 and 2). On the basis of differential expression levels for the mRNA fragments on the agarose gels, 12 genes were up-regulated in the AI group (Fig. 3) and the functional roles and sequence similarities are summarized in Table 4. BLAST searches in the NCBI GenBank revealed that the DEGs shared similarities (83–100%) with sequences from the canine species.

**Table 4:** Identity of DEG sequences by BLAST searches.

DEG No.	GeneBank accession No.	Identity	Base pair sequenced	Homology^a^
1	XM_843567.1	Canis familiaris similar to nuclease sensitive element binding protein 1 (LOC607025), mRNA	168	85/85 (100%)
2	XM_849493.1	Canis familiaris similar to G1/S-specific cyclin D2 (LOC611782), mRNA	385	203/205 (99%)
3	XM_533724.2	Canis familiaris similar to cellular nucleic acid binding protein 1, transcript variant 1 (LOC476518), mRNA	819	416/417 (99%)
4	AC186216.7	Canis familiaris chromosome 31, clone XX-152G22, complete sequence	98	55/57 (96%)
5	AC190102.9	Canis familiaris chromosome 26, clone XX-195P12, complete sequence	58	32/33 (96%)
6	XM_861618.1	Canis familiaris similar to ribosomal protein P1 isoform 2, transcript variant 4 (LOC478356), mRNA	521	302/304 (99%)
7	EF568723.1	Canis lupus familiaris voucher AG08103 cytochrome oxidase subunit I (COXI) gene, partial cds; mitochondrial	283	147/179 (83%)
8	XM_845806.1	Canis familiaris similar to 60S ribosomal protein L26 (Silica-induced gene 20 protein) (SIG-20) (LOC608703), mRNA	494	261/264 (98%)
9	XM_533197.2	Canis familiaris similar to 60S acidic ribosomal protein P2 (LOC475991), mRNA	392	198/198 (100%)
10	AB499820.1	Canis lupus mitochondrial DNA, complete genome, haplotype: Jw235	406	235/240 (97%)
11	HM048871.1	Canis lupus familiaris breed Tibetan Mastiff mitochondrion, complete genome	287	169/177 (95%)
12	XM_850384.1	Canis familiaris similar to small nuclear ribonucleoprotein polypeptide G (LOC612653), mRNA	497	325/327 (99%)

a Percentages based on BLAST searches of the GenBank database and the number of base pairs (query/subjected) that were compared.

### Confirmation of differentially expressed genes by real-time RT-PCR

To confirm the results of ACP RT-PCR, we performed real-time RT-PCR analysis for DEG expression using cDNA from AI and PA fetuses. Three DEGs were selected, G1/S-specific cyclin D2 (Cyclin D2), cytochrome oxidase subunit I (COI) and nuclease sensitive element binding protein 1 (NSEBP1), and their quantitative expression patterns are presented in Fig. 3. The quantitative PCR analysis revealed that transcripts of these selected genes had similar expression patterns which are in agreement with the ACP-based RT-PCR.

Since delayed fetal development was evident in the canine parthenotes, we examined expression levels of apoptosis-related genes (BAX, BCL2, and BCL2L1) by real-time PCR. The expression levels in the PA group were similar to the AI group, except for reduced expression of BAX and BCL2 in the day 30 canine parthenotes (P<0.05) and increased expression of BCL2L1 in the day 32 canine parthenotes (P<0.05, Fig. 3B). The day 32 parthenotes demonstrated a significantly higher BAX/BCL2 ratio than their AI counterparts (P<0.05).

### Organotypic development and regulation of FADD and caspase 8 gene expression in canine parthenotes

**Figure 4:**
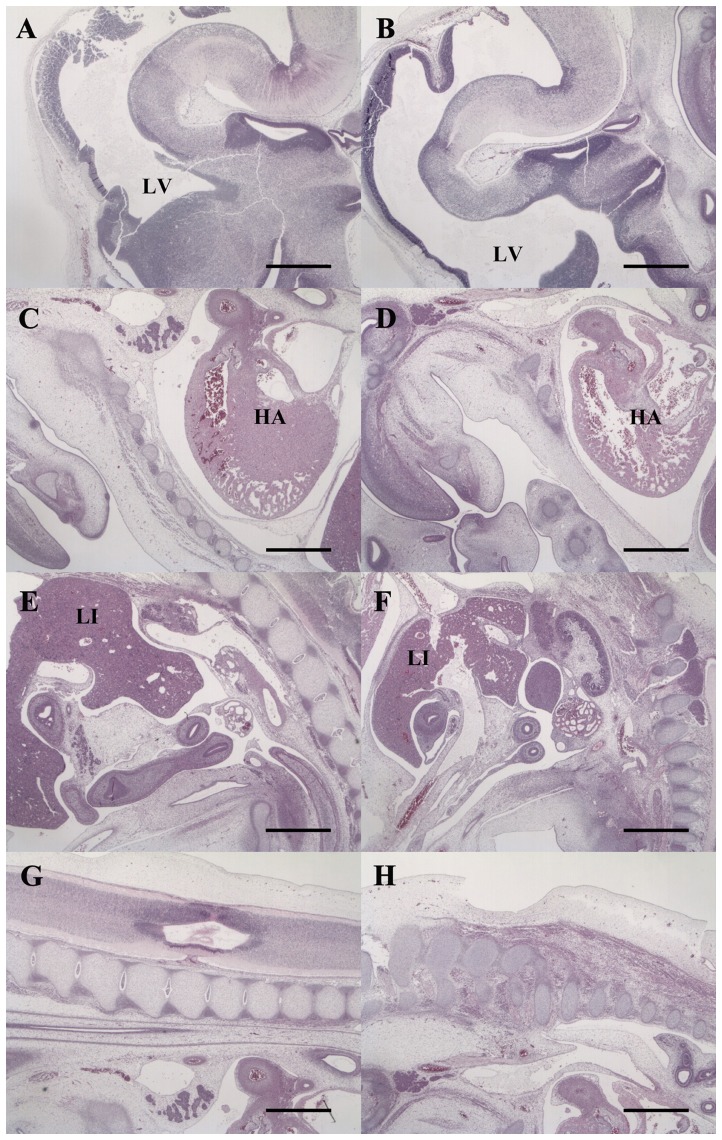
Histological analysis of day 30 control (A, C, E, G) and parthenogenetic (B, D, F, H) canine fetuses. H&E staining of head (A, B), heart (C, D), liver and intestine (E, F), and vertebral area (G, H) showed similar development of major organs in canine parthenotes and controls. Note also the artificially inseminated fetus on day 30 was slightly larger than the canine parthenote. All scale bars represent 1 mm. LV, lateral ventricle; HA, heart; LI, liver.

**Figure 5:**
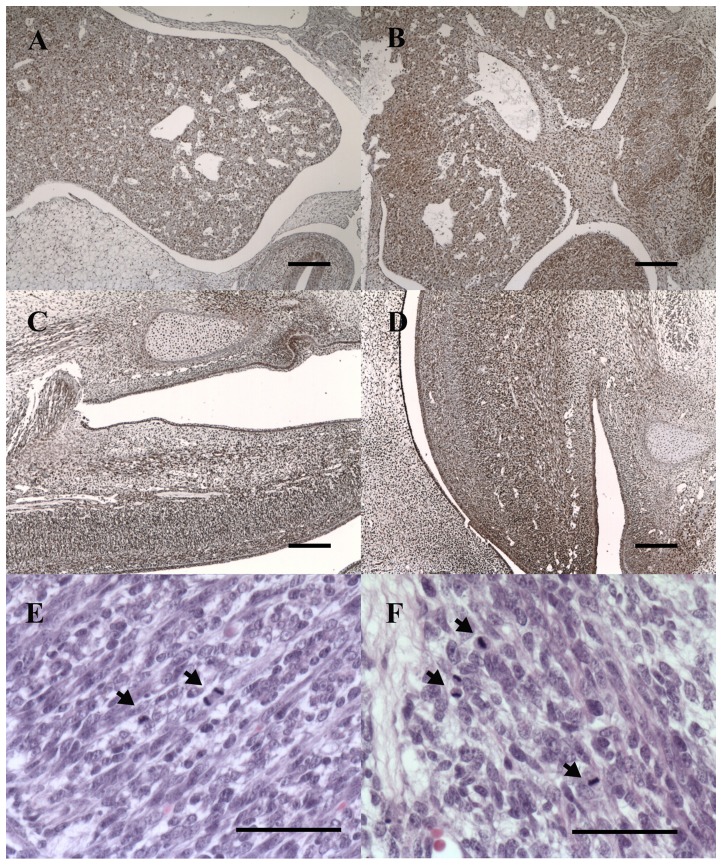
Immunohistochemical stain of PCNA in control and parthenogentic canine fetuses. PCNA staining for proliferating cells in day 30 control (A, C) and parthenogenetic (B, D) canine fetuses, demonstrating similar distribution patterns of dark brown PCNA-positive cells in regions of fetal liver (A, B) and brain (C, D). Day 30 parthenote (F) showed a similar level of mitotic figures (arrows) to those of control (E). Scale bars in A–D, 200 µm. Scale bars in E–F, 50 µm.

Histological analysis did not reveal any organotypic abnormalities in the day 30 canine parthenote (Fig. 4). The canine parthenote fetus showed qualitatively similar development of the major organs including heart, liver, intestine and vertebrae to the same gestational age controls except that all the organs were smaller. The expression of PCNA was localized using immunohistochemisty for assessing cell proliferation and fetal development in AI and PA fetuses. The day 30 canine parthenote and a control fetus demonstrated similar distribution patterns of cell proliferation in liver and brain as shown in Fig. 5, as well as in other fetal tissues (data not shown). Also, mitotic figures in the day 30 canine parthenote occurred at a similar frequency to those in the control (Fig. 5. E, F).

**Figure 6:**
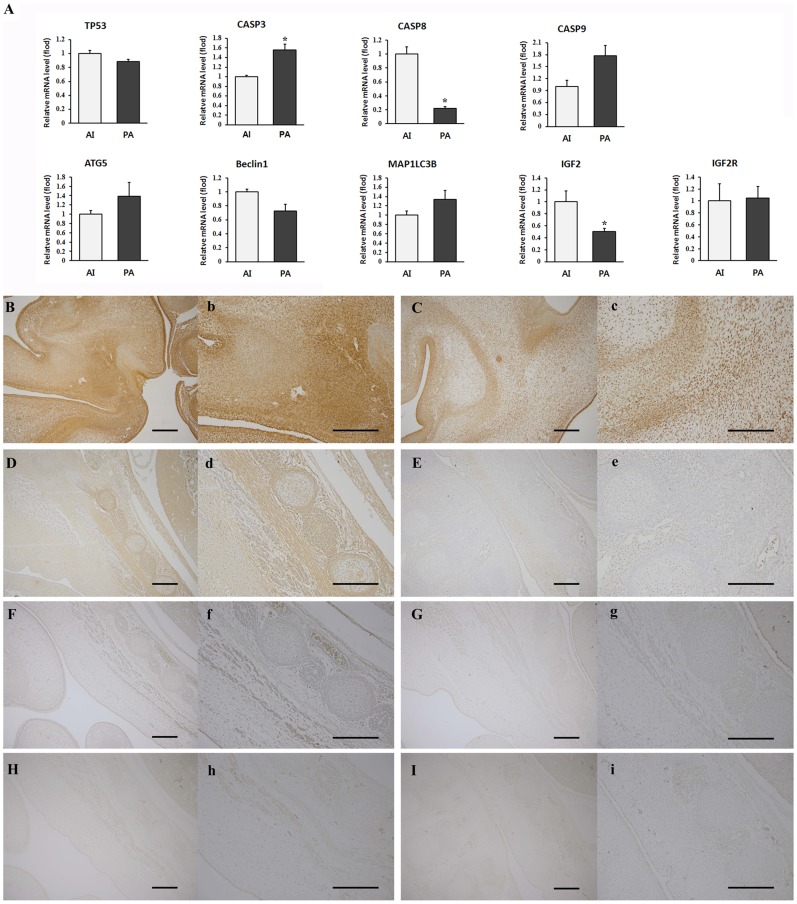
Comparison of expression patterns for apoptosis, autophage and growth related genes in day 30 controls and canine parthenotes. (A) Gene expression levels of apoptosis, autophage and growth related genes. Data presented as mean ± SEM of at least three replicates. The asterisk denotes a probability value of <0.05. (B–H, b–h) Immunohistochemical analysis of control (B, D, F, H, b, d, f, h) and canine parthenogenetic fetuses (C, E, G, I, c, e, g, i). Immunohistochemacal staining for CASP8 (B–C, b–c), FADD (D–E, d–e), LC3A/B (F–G, f–g) and ERK1 (H–I, h–i) were performed and canine parthenote exhibited decreased expression of CASP8 and FADD, while no differences in LC3A/B and ERK1 were identified. All scale bars represent 200 µm.

Cell proliferation patterns in parthenote were relatively similar to control while the apoptosis related genes showed altered expression levels, so we further assessed the expression levels of apoptosis (TP53, CASP3, CASP8, CASP9), autophage (ATG5, BECN1, MAP1LC3B) and growth-related genes (IGF2, IGF2R) in both groups by real-time RT-PCR (Fig. 6A). Compared to the control, the day 30 canine parthenote exhibited significantly higher CASP3 mRNA expression, although CASP8 mRNA expression was significantly decreased (P<0.05). The levels of TP53, CASP9, ATG5, BECN1 and MAP1LC3B expression in the parthenote were not significantly different from those in the control fetus. No significant difference in IGF2R expression was observed between the two groups but the expression of IGF2 was considerably lower in parthenotes (P<0.05).

To examine these results in more detail, we evaluated the expression levels of FADD, CASP8, LC3A/B and ERK1 protein in parthenote and control by immunohistochemistry. Correlated with mRNA expression levels, canine parthenote exhibited decreased expression of CASP8 and FADD, while no differences in LC3A/B and ERK1 were identified.

## Discussion

Although *in vivo* development of PA embryos has been reported in mammals [16], [17], [18], little information is available on their developmental characteristics and there are no reports on post-implantation development of PA activated canine oocytes. In the present study, canine parthenotes were able to develop to the stages of limb-bud formation, but it was developmentally delayed with different gene expression patterns compared to the biparental counterparts.

The results demonstrated that nine out of ten recipients that received PA canine embryos became pregnant (Table 3). Although successful cloning of dogs was first reported in 2005 [32], conventional assisted reproductive technologies such as *in vitro* oocyte maturation, fertilization and embryo development are not available in dogs due to lack of efficient protocols [40], [41], [42]. Because of this, knowledge of preimplantation events in this species is very limited. Therefore, the success of contemporary protocols for activating dog oocytes as judged by their ability to mimic the events that occur during normal fertilization was assessed by studying post-implantation viability of transferred parthenogenetic embryos. The combined use of a Ca^2+^ stimulating substance with an inhibitor of protein synthesis has been widely used for activation of domestic animal oocytes [43] and the treatment of calcium ionophore with a phosphatase inhibitor, 6-dimethylaminopurine, was effectively triggered activation of canine oocytes. Although the proportion of transferred PA embryos that implanted was significantly lower than in the AI group, the pregnancy rate of recipient dogs in the PA group was similar to the AI group and, furthermore, it was markedly higher than the results from transferring canine SCNT embryos [30], [31], [32], [33], [44], [45]. Only one attempt failed to result in pregnancy, but no decision can be drawn concerning the effect of oocyte stage or number of transferred embryos on *in vivo* viability due to the limited number of experimental trials. These results indicated that the methods used in this study for chemically activating canine oocytes and the embryo transfer protocols were appropriate for supporting further development of canine embryos.

After successful development of PA canine embryos up to post-implantation stages was confirmed, we compared their development to their *in vivo* counterparts. In agreement with previous studies conducted in other species, canine parthenotes were able to develop to the stages of beating heart and limb-bud formation [16], [17], [18], [46], [47]. Also, it is assumed that canine parthenotes will not develop to term and their death probably occurred around day 32 of pregnancy since the general growth of PA embryos and their trophoblastic tissue was delayed and the total weight of placental sacs and fetuses on day 32 was lower than those of day 30 (Fig. 1). The abnormal placental developments from the early stages after implantation suggest the disturbed embryo-maternal communication during the peri-implantation period [48]. Accordingly, many critical physiological events occur during the development of AI and PA fetuses, and we applied ACP-based RT-PCR analysis to help understanding of molecular mechanisms underlying canine embryonic development. With this technique, we identified 12 DEGs that are prominently expressed in AI fetuses compared to PA fetuses. We selected 3 genes based on the sequencing results to assess changes in expression patterns during days 28 to 32 of gestation by using quantitative RT-PCR. We observed significantly higher expression levels of Cyclin D2 and COI in the AI group compared to the PA group, while the transcription level of NSEBP1 showed no significant difference. The cyclins play a central role in the control of cell proliferation and form a sensor that connects intracellular cell cycle machinery to external signals [49]. Since it has been reported that cyclin D2 is up-regulated at gastrulation and is exquisitely regulated during gastrulation and neurulation [50], the different expression pattern of cyclin D2 between the PA and AI groups are likely related to the difference in viability of those fetuses. Transcription levels of mitochondrial DNA are important in the regulation of mitochondrial oxidative capacity and may be linked to oxidative metabolism and energy demand [51]. In the present study, the expression level of COI was reduced in the PA group compared to the AI group (Fig. 3), thus, these genes may have functions associated with normal embryo development and fetal survival.

For further evaluation of the differences between AI and PA fetuses, we analyzed the expression patterns of genes related to the apoptosis signaling pathway: BAX, BCL2, and BCL2L1. The Bcl2 family is central to the regulation and execution of apoptosis, and is subdivided into pro-apoptotic (for example Bax and Bad) and anti-apoptotic members (for example Bcl2 and Bcl2L1), which can form homo- and hetero-dimers [52]. Therefore, the ratio between pro- and anti-apoptosis proteins is thought to be important in determining the resistance of a cell to apoptosis [52], [53]. In the present study, the PA group exhibited a significantly higher BAX/BCL2 ratio than the AI group, so it can be inferred that higher apoptosis was occurring on day 32 of the canine parthenotes. It has been widely accepted that coordination between cell growth and death is a fundamental requirement for embryogenesis, organ metamorphosis and tissue homeostasis [53], [54], hence, incongruity of expression patterns in developmentally important genes could be one of the reasons for developmental retardation in canine parthenotes.

On the other hand, in the histological analysis and PCNA immunohistochemistry, the well-developed canine parthenote demonstrated similar fetal growth characteristics to the AI control, such as formation of major organs without organotypic abnormalities and similar patterns of cellular proliferation (Figs. 4 and 5). In agreement with our observations, ovine PA fetuses at day 21 of pregnancy were viable with beating hearts and showed no differences in fetal membrane morphology or in successful development of major organs [17]. Therefore, canine parthenotes could survive through early fetal development in the absence of a paternal genome, however, the apoptosis and cell cycle-related genes showed altered expression. Consequently, we evaluated the expression patterns of genes involved in apoptosis, autophage and cell proliferation to provide more detailed information on mechanisms of developmental retardation and cessation of parthenote viability. As shown in Fig. 6, the expression of two apoptosis related genes, CASP3 and CASP8, was significantly altered in the day 30 PA fetuses compared to control. To elucidate the reliability of the real time PCR data and to understand the interaction of caspase with upstream signaling molecules, we conducted immunostaining for CASP8 and FADD. The day 30 PA fetus showed relatively low expression of CASP8 and FADD proteins, suggesting that lower expression of CASP8 occurred following the down-regulation of FADD. It is well known that precise regulation of apoptosis is important for normal, functional development since it is necessary for the elimination of unwanted cells with potentially harmful mutations [55], [56]. The caspases form a caspase-cascade system that plays a central role in the induction, transduction and amplification of intracellular apoptotic signals, and CASP8 is called the initiator of apoptosis caspases [48]. The FADD is an adaptor for relaying apoptotic signals initiated by death receptors and it contains the death effecter domain that binds to the pro-domain of CASP8 [57]. Interestingly, gene-targeting studies have revealed that FADD and/or CASP8 deficiency results in early embryonic lethality, indicating that these factors are essential for embryonic development [58], [59]. Accordingly, our results provide evidence that down-regulation of CASP8 and FADD may produce alterations in cellular processes and lead to developmental failure of canine parthenote.

No significant difference in autophage-related genes or IGF2R expression was observed between the two groups but the expression of IGF2 was considerably lower in parthenote, suggesting that canine IGF2 might feature imprinted genes that are expressed from the paternal genome; lacking developmentally important gene products may also be a cause of developmental abnormalities in canine PA embryo [12]. Genomic imprinting is a method of gene regulation to either express or repress the gene in accordance with its parental origin, and many of these imprinted genes have important roles in development [35]. Several studies have been conducted to identify imprinted genes because altered dynamics of imprinting can lead to a range of developmental consequences, and aberrant imprinting has been implicated in many diseases, yet it has been studied in relatively few mammalian species [9], [60]. The dog (*Canis familiaris*) is considered valuable as a model organism, because of the availability of extensive, high quality genome sequences and the exhibition of a wide range of diseases similar to those of humans [60], [61], so, canine PA fetuses can be valuable for the comparative study of genomic imprinting.

In this study, we report the first successful development of PA canine embryos through post-implantation stages. This study confirmed that the protocols used are suitable for activating canine oocytes artificially and can support viability and the developmental potential of canine embryos. In addition, we demonstrated the expression pattern of several genes, including cell cycle regulating-, mitochondria-related and apoptosis-related genes that were different between parthenotes and AI counterparts. Our results indicate that studies on development of canine PA fetuses will provide insights into the molecular mechanisms involved in the respective roles of paternal and maternal genomes during mammalian development as well as providing useful tools for screening imprinted genes vital for embryo development.
