# Correction: Altered Cell Cycle Gene Expression and Apoptosis in Post-Implantation Dog Parthenotes

**DOI:** 10.1371/journal.pone.0277164

**Published:** 2022-11-01

**Authors:** Jung Eun Park, Min Jung Kim, Seung Kwon Ha, So Gun Hong, Hyun Ju Oh, Geon A. Kim, Eun Jung Park, Jung Taek Kang, Islam M. Saadeldin, Goo Jang, Byeong Chun Lee

After this article [1] was published, concerns were raised about the source of the animals used in experiments for this article, and about welfare considerations for the animals while in the laboratory. In response to queries about these experiments, this notice provides additional information regarding the animal care, housing, sourcing, socialisation, exercise, surgical procedures, and heterogeneity of the animals:

- The animals used in this study were provided from a breeding facility, Kumsung Animal supply, located in Chungcheongnam-do, South Korea, which was not an accredited animal facility for scientific research, but the facility was maintained as a research animal provider.
- In this study, naturally ovulated and matured oocytes were recovered from donor female dogs and transferred to recipient females. Animal handling procedures such as pre-anesthesia treatment, anesthesia, and surgical operations were performed, and post-surgery veterinary care such as administration of antibiotics, analgesics, and fluids, were provided to the research animals by a licensed veterinarian.
- The experimental procedure could be less invasively performed in larger sized dogs as the reproductive organ size is proportional to the size of the animal and larger reproductive organs provide better accessibility to the surgical site for experimental procedures. Larger sized dogs ovulate a greater number of oocytes per reproductive cycle and thus, more oocytes are obtained from fewer animals.
- The experiment was conducted on both nulliparous and multiparous healthy females and the results were analysed with pooled data acquired from both groups.
- 124 oocytes were collected from 14 oocyte donors and 123 parthenogenetically activated embryos were transferred to 10 recipients. 3 females were used for the control group to obtain conceptuses derived from fertilisation. The total number of females used in this study was 27 (14 oocyte donor dogs and 10 recipients in the experimental group, 3 females in the control group). The samples were collected at 3 different gestational days, 28, 30 and 32 days of pregnancy, to analyse temporal change patterns in fetal/placental growth and gene expressions during development.
- Different experimental dogs were used for oocyte donors and recipients. In the case of recipient dogs, the surgical procedure was performed for embryo transfer and conceptus retrieval procedure 30 to 32 days after the previous surgery for embryo transfer. Surgical wounds were completely healed 30–32 days after the initial surgery and a mid-line ventral incision was made to expose the reproductive tract. Reproductive organs of recipient females were surgically removed by ovariohysterectomy and spayed females received post-operation treatment and recovered after surgery.
- Anesthesia was induced with 6 gm/kg ketamine HCl and 1 mg/kg xylazine, and general anesthesia was maintained with 2% isoflurane.
- The first day of spontaneous estrus was determined by daily visual examination for vulvar swelling and serosanguinous discharge. Blood samples (3–5 ml) were collected and serum progesterone concentrations were determined with a DSL-3900 Active progesterone Coated-Tube Radioimmunoassay kit (Diagnostic Systems Laboratories, Inc., TX, USA).
- No euthanasia was performed on the oocyte donors nor recipient females. There were no incidences of wound dehiscence, intercurrent infections, illness, or mortalities. At the end of experiments, fully recovered healthy animals were returned to the animal provider and subsequent care was provided by the facility. The animals were not used in other research projects either before or after the experiments in article [1].
- Commercially available, nutrient balanced dog food was provided daily with water ad libitum and commercial dog snacks were provided for environmental enrichment. All food was stored to prevent contamination and deterioration of its nutritive value. Foods requiring refrigeration were stored accordingly.
- The experimental animals were individually housed for up to one month in units of several 1.12 m^2^ cages that were separated with adjustable walls. The adjustable walls were removed to combine between two and four units per dog depending on the size of the dog to create individual separated spaces for each animal. Additional floor space was calculated as follows: (length of dogs in inches + 6) x (length of the dog in inches +6) = additional floor space in square inches. The interior height of each primary enclosure was greater than 6 inches higher than the head of the tallest dog when in a normal standing position. The exact dimensions of individual primary spaces are no longer available.
- Since the research animals used in this study were either in estrus or in the immediate post-operative stage they were individually housed to provide careful post-operation treatment after the surgical procedures. They did not play together while in estrus and while their incisions were healing. Routine veterinary checks were performed at least three times per day by a licensed veterinarian and veterinary technicians. In addition to social interaction with veterinarians and staff, dogs were provided with chew toys.
- The animal housing facilities contained animals securely, restricted other animals from entering and protected the animals from injury.

The *PLOS ONE* Editors consulted with experts in animal welfare and animal research methodology who assessed the article and the authors’ comments. They noted that the enclosure heights and individual housing are not good practice for longer periods (e.g. days) and the justification given for post-operative separation and for separation of dogs in estrus is not persuasive. They also raised concerns that the article and the comments provided in post-publication discussions did not report the use of post-operative analgesia following any of the procedures. However, the consulting experts stated that, overall, it appears that the care, housing, sourcing and exercise regimes, while not best practice, complied with many of the standards in force at the time for research with dogs.
